# Chemoresistance: Intricate Interplay Between Breast Tumor Cells and Adipocytes in the Tumor Microenvironment

**DOI:** 10.3389/fendo.2018.00758

**Published:** 2018-12-11

**Authors:** Ilze Mentoor, Anna-Mart Engelbrecht, Paul J. van Jaarsveld, Theo Nell

**Affiliations:** ^1^Department of Physiological Sciences, Faculty of Science, Stellenbosch University, Stellenbosch, South Africa; ^2^Non-Communicable Diseases Research Unit, South African Medical Research Council, Cape Town, South Africa; ^3^Division of Medical Physiology, Faculty of Medicine and Health Sciences, Stellenbosch University, Stellenbosch, South Africa

**Keywords:** obesity, adipose tissue, breast cancer, inflammation, treatment resistance

## Abstract

Excess adipose tissue is a hallmark of an overweight and/or obese state as well as a primary risk factor for breast cancer development and progression. In an overweight/obese state adipose tissue becomes dysfunctional due to rapid hypertrophy, hyperplasia, and immune cell infiltration which is associated with sustained low-grade inflammation originating from dysfunctional adipokine synthesis. Evidence also supports the role of excess adipose tissue (overweight/obesity) as a casual factor for the development of chemotherapeutic drug resistance. Obesity-mediated effects/modifications may contribute to chemotherapeutic drug resistance by altering drug pharmacokinetics, inducing chronic inflammation, as well as altering tumor-associated adipocyte adipokine secretion. Adipocytes in the breast tumor microenvironment enhance breast tumor cell survival and decrease the efficacy of chemotherapeutic agents, resulting in chemotherapeutic resistance. A well-know chemotherapeutic agent, doxorubicin, has shown to negatively impact adipose tissue homeostasis, affecting adipose tissue/adipocyte functionality and storage. Here, it is implied that doxorubicin disrupts adipose tissue homeostasis affecting the functionality of adipose tissue/adipocytes. Although evidence on the effects of doxorubicin on adipose tissue/adipocytes under obesogenic conditions are lacking, this narrative review explores the potential role of obesity in breast cancer progression and treatment resistance with inflammation as an underlying mechanism.

## Introduction

Breast cancer continues to be a major health risk for women globally (1, 2). Lifestyle-related risk factors including overweight and obesity (adiposity) have reached epidemic proportions (3, 4), and are considered major risk factors for breast cancer development and progression (5).

Adipose tissue plays an important physiological role as a metabolically active storage compartment and endocrine organ due to its diverse ability to secrete various adipokines (6). Adipose tissue dysfunction in relation to obesity has been linked to accelerated growth and the survival of breast cancer cells (7, 8).

Adipose tissue dysfunction is mainly characterized by inflammation which is primarily mediated by rapid adipose tissue remodeling (hypertrophy and hyperplasia) (9). This results in dysfunctional synthesis of several adipokines in coordination with immune cell infiltration leading to a state of sustained low-grade inflammation, which activates downstream signaling pathways favoring cancer cell survival (increased proliferation and decreased apoptosis) and hence contributing to cancer progression and metastasis (10–12).

Furthermore, adipose tissue and/or adipocytes in the tumor microenvironment serve as an exogenous energy source for the survival of breast cancer cells (13, 14), especially since adipose tissue is abundant in the breast (15). It is further proposed that breast cancer cells modulate lipid metabolism by altering the secretion of adipokines through adipocytes, resulting in the release of free fatty acid (FFA) providing energy substrates, that cancer cells need to sustain its high proliferation demand (13).

Pre-clinical evidence highlights obesity as a key player in breast cancer chemotherapeutic drug resistance (16–19). This finding bear's great clinical significance for overweight/obese breast cancer patients being treated with chemotherapeutic agents such as doxorubicin (20), since, obese and normal weight patients receive the same treatment regimen (21). Studies have also confirmed this in showing that obesity is associated with poor clinical outcomes in breast cancer patients treated with chemotherapeutic agents including doxorubicin (22, 23).

Doxorubicin is a highly sensitive alkylating antineoplastic agent used as a first line adjuvant regimen for breast cancer patients (24), despite its high sensitivity as a chemotherapeutic agent, it is also associated with a diverse range of cellular toxicities and the development of treatment resistance (25). Additionally, doxorubicin also negatively impacts on adipose tissue function (26–29). This is of clinical significance since obesity is associated with an increased risk for various types of cancers being treated with doxorubicin (20). However, few studies exist in which the effects of doxorubicin on adipose tissue in the context of obesity and dysfunctional adipose tissue is investigated. We proposed that using doxorubicin treatment on dysfunctional adipose tissue and/or adipocytes, may exacerbate the negative effects of obesity *per se*, and further dysregulate adipokine secretion.

It is imperative to explore and understand the cellular mechanisms whereby obesity negatively affects chemotherapy outcomes. Identifying molecular mechanisms in which doxorubicin affect adipose tissue could contribute in describing molecular mechanisms and identifying potential novel pharmacologic targets and development of the appropriate management protocols of doxorubicin related toxicities in order to improve over-all survival of these cancer patients. This narrative review will mainly focus on (i) the *pathological links between adiposity and breast cancer in the context of inflammation as an underlying mechanism* and, (ii) *the role of adiposity in breast cancer treatment (doxorubicin) resistance and the possible mechanisms that contribute to treatment resistance*.

## Adiposity and Breast Cancer

Globally, the increasing burden of breast cancer is considered the second most prevalent cancer diagnosed amongst women (1, 30) in both developed and developing countries (2, 31). Estimations rank breast cancer as the fifth leading cause of death globally at 626,679 deaths per annum (1, 30).

Despite many efforts to reduce cancer mortality by implementing lifestyle-related modifications, limited progress has been made due to the very complicated interplay between dietary behaviors and other lifestyle modifications (32, 33). This is especially problematic since recent epidemiological studies strongly suggested that adiposity (excess adipose tissue) is considered a significant risk factor in many lifestyle-associated cancers including breast cancer (34–37).

### Adipose Tissue Is a Complex Functional Tissue

Fundamentally, adipose tissue is a complex and important endocrine organ impacting various physiological systems (38). It functions as both an energy storage compartment and a metabolic active endocrine organ (6), secreting various bioactive substances (pro- and anti-inflammatory) known as adipokines (39), including but not limited to leptin, adiponectin (Apn), tumor necrosis factor-alpha (TNF-α), interleukin-1β (IL-1β), interleukin-6 (IL-6), resistin and macrophage chemoattractant protein-1 (MCP-1) (40, 41). Additionally, adipose tissue also plays a functional role in steroid sex hormone and growth factor production, and is integral in the development of insulin resistance, hyperglycaemia and breast cancer (42, 43). Epidemiological and experimental models support the role of a dysregulation in adipokine synthesis and their actions in relation to adiposity and adipose tissue dysfunction, to the development of various disease states, including breast cancer (5, 44–51).

### Dysfunctional Adipose Tissue, Inflammation, and Breast Cancer

**Figure 1:**
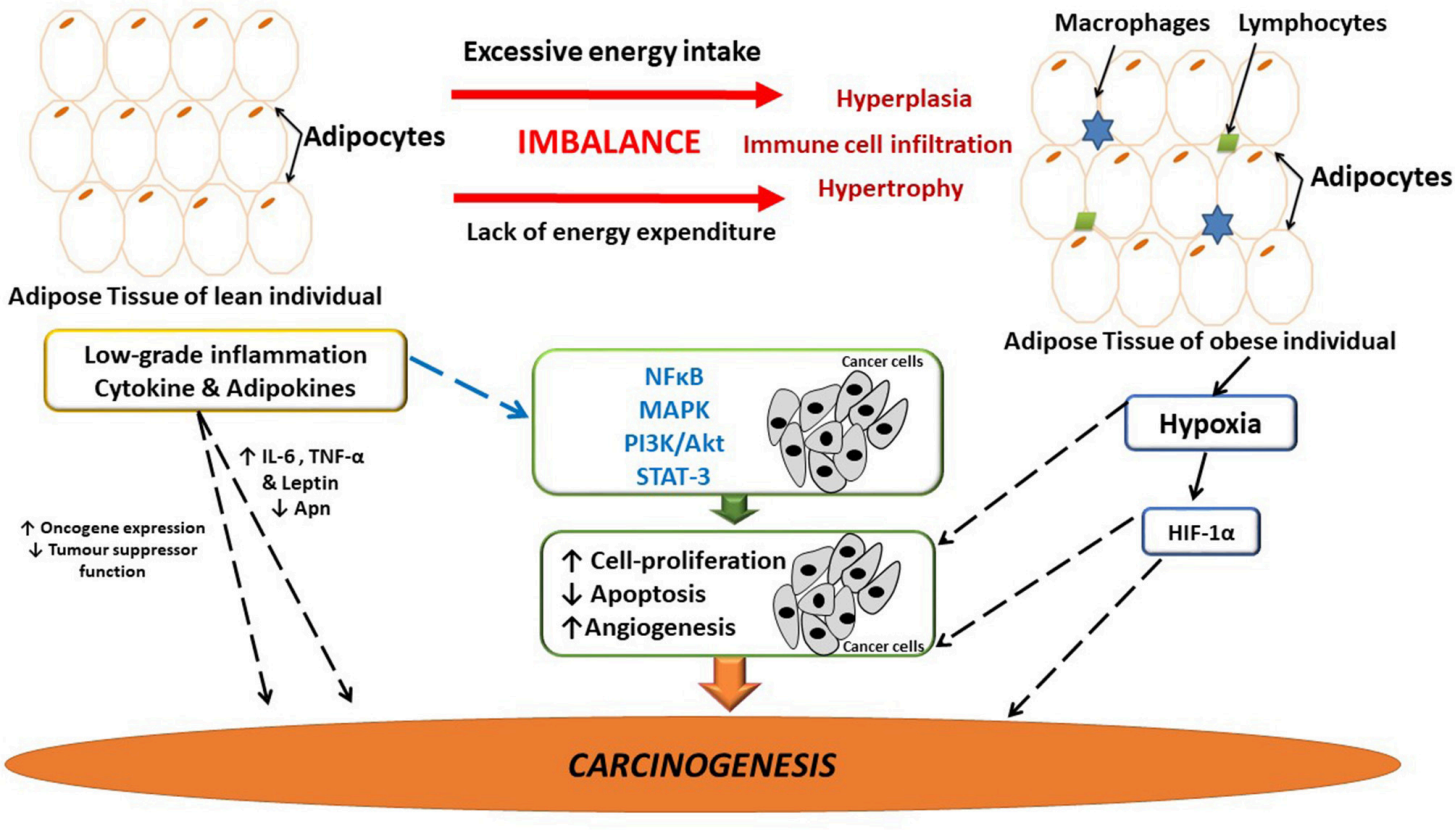
The link between adipose-induced inflammation and cancer. Adipose tissue dysfunction is associated with sustained low-grade inflammation and it may be linked to breast cancer development and progression. Several inflammatory mediators are implicated in tumor development and progression. Possibly as a result of the sustained inflammatory signaling having downstream effects on major pathways involved in angiogenesis, cell-proliferation and apoptosis, thus having the ability to influence carcinogenesis. Hypoxia in adipose tissue also induces the release of inflammatory mediators, thus further exacerbating inflammation. Apn, adiponectin; IL-6, interleukin-6; HIF-1α, hypoxia inducible factor-1α; MAPK, mitogen activated protein kinase; NFκB, nuclear factor kappa B; PI3K/Akt, phosphoinositide-3-kinase; STAT-3, Signal transducer and activator of transcription-3; TNF-α, tumor necrosis factor-α.

Dysfunctional adipose tissue is characterized by low grade inflammation, primarily mediated by rapid hypertrophy/hyperplasia (adipose tissue remodeling) as well as immune cell infiltration (52), resulting in the deregulated synthesis of several adipokines i.e., IL-1β, IL-6, interleukin-8 (IL-8), resistin, leptin and MCP-1 (7, 8, 15, 53–55) (Figure 1). These inflammatory mediators attract monocytes (differentiated into macrophages) and T-lymphocytes, stimulating the synthesis of both pro-inflammatory and pro-angiogenic factors (56), collectively contributing to a chronic cycle that sustains an inflammatory *milieu*. Increased IL-6 and leptin levels has been shown to supress 5′ adenosine monophosphate-activated protein kinase (AMPK), well-known for its' anti-inflammatory effects in adipose tissue (57). Additionally, adipose tissue-induced inflammation also attenuates the suppression of nuclear factor kappa B (NFκB), p65 phosphorylation and also induces M1 to M2 macrophage phenotype switching. The latter is due to the pro-inflammatory state in which saturated fatty acids bind to the toll-like receptors on macrophages (58) and upregulate the secretion of various pro-inflammatory mediators (IL-1β and TNF-α) in adipose tissue (59, 60), creating a state of chronic systemic low-grade inflammation.

Inflammation is a well-known predisposing risk factor for tumorigenesis and a hallmark of cancer (61, 62). Pre-existing pro-inflammatory microenvironments are associated with an increased risk for cancer, as in the case for inflammatory breast cancer (63). Remarkable similarities exist between dysfunctional adipose tissue and the tumor microenvironment where infiltration of immune cells initiate the secretion of pro-inflammatory molecules, thereby sustaining and promoting the progression of both obesity and breast cancer (64) (Figure 1).

The complex pathophysiology that exists between adipose tissue, inflammation and breast cancer involves inflammatory mediators (i.e., IL-6 and TNF-α) that enhances tumor progression and survival (65). Persistent inflammatory signaling (intracellular NFκB) induces downstream effects on major biochemical pathways affecting carcinogenesis (Figure 1). For example, the mitogen activated protein kinase (MAPK) family modulates cellular proliferation *via* the phosphoinositide-3-kinase (PI3K/Akt), and the MAPK pathways which regulates and affects mitogenic, anti-apoptotic as well as pro-angiogenic effects (60). Moreover, IL-6 secreted by adipose tissue binds to IL-6 receptor on breast cancer cells and activates the Janus family of kinases that phosphorylates signal transducer and activator of transcription-3 (STAT-3) (66). These events induce the expression of pro-survival genes (i.e., *bcl-x*) (67), characteristic of a pro-carcinogenic state promoting breast cancer cell survival and proliferation (Figure 1).

Obesity-induced cytokine secretions are detected in local adipose tissue and serum (68). These elevated circulating cytokines (IL-6, IL-8, TNF-α, and vascular endothelial growth factor; VEGF), exert effects at distant sites (69), that can promote breast cancer development through upregulation of inflammatory mediator synthesis and increased immune cell infiltration as well as angiogenesis (70, 71). Additionally, overweight/obese patients display a large number of crown-like-structures (necrotic adipocytes surrounded by immune cells) in mammary adipose tissue compared to normal weight breast cancer patients. These crown-like structures are characteristic of local inflammation (66, 72), and associated with an upregulation of pro-inflammatory cytokines and aromatase expression (73). Although the role of cytokines in obesity and breast cancer development have been reported, the effects of other adipokines should be considered as a possible relationship between obesity and the development of breast cancer.

Leptin and adiponectin (Apn) have been antagonistically implicated for their roles in inflammation and tumorigenesis (Figure 1) (74). Leptin increases the synthesis of pro-inflammatory cytokines and plays a role in breast cancer development by increasing cellular proliferation and angiogenesis (75). Elevated serum leptin levels and increased expression of leptin receptors is also reported in breast cancer patients that is often associated with higher pathological grade tumors and cancer treatment resistance (76, 77). Adiponectin is decreased in obese patients, the metabolic syndrome as well as in breast cancer patients thus lowering the risk of cancer development due to an upregulation of apoptosis and its' anti-inflammatory properties (78–81).

Excess adipose tissue (overweight/obesity) is also associated with increased secretion of insulin-like growth factor-1 (IGF-1) in breast, colon, lung and prostate cancer patients (82). The over expression of insulin-like growth factor-1-receptor (IGF-1R) was observed in both breast and pancreatic tumor tissue (83), where inhibition of apoptosis and stimulation of cellular proliferation *via* the PI3K-AKT-mTOR and RAS/Raf/MEK pathways are implicated (83).

Additionally, loss of tumor suppressor function, increased cell cycling and stimulation of oncogenes also promote inflammation and exacerbates inflammatory related signaling pathways (69, 84, 85) (Figure 1). For example, the p53 gene mutation promotes inflammation in the tumor microenvironment by inducing the synthesis of IL-1, IL-6, TNF-α, and activates NFκB (86, 87), which maintains inflammation in the tumor microenvironment and enhances genomic instability (88, 89). In addition, p53 has also been shown to induce the PI3k/Akt/mTOR pathway, which can induce the synthesis of pro-inflammatory mediators (90). As a result of rapid hypertrophy and hyperplasia (91) (Figure 1), hypoxia inducible factor-1α (HIF-1α) is upregulated (92), which binds to transcription factors on VEGF and angiopoietin-2 target genes, stimulating angiogenesis in the microenvironment, which is also known to exacerbate local inflammation (92).

Others report that obesity-induced inflammation may also play a role in breast cancer tumor invasion and metastasis. Here, epithelial mesenchymal transition (EMT) can be induced by various pro-inflammatory markers, i.e., IL-6, IL-8, TNF-α, and CCL2 derived from cancer-associated adipocytes (93–95).

It is evident from these findings that obesity is a factor casual in the development of breast cancer, involving molecular mechanisms in relation to inflammation, immune cell infiltration and adipokine dysfunction. Supporting evidence includes obesity as a negative prognostic factor for breast cancer independent of menopausal status, tumor stage, and tumor hormone–binding characteristics (96, 97).

## Breast Cancer Treatment

Chemotherapy still remains one of the conventional treatment options in addition to radiotherapy and surgery, which significantly improves cancer patients' overall-survival (98, 99). Several chemotherapeutic drug classes exist which are associated with beneficial clinical outcomes for cancer patients (100).

Doxorubicin, also known as Adriamycin or hydroxyl daunorubicin (101), is classified as an anthracycline antibiotic, exhibiting broad-spectrum anti-neoplastic activity (24, 101), and is used to treat a range of malignancies of the breast (used as first line adjuvant chemotherapeutic agent), bladder, stomach, lung, ovaries, thyroid as well as multiple myeloma, Hodgkin- and non-Hodgkin's lymphoma, due to poor tumor selectivity (102).

Doxorubicin interacts with deoxyribonucleic acid (DNA) by intercalation, thereby inhibiting macromolecule biosynthesis (103). This inhibits topoisomerase II (DNA repair function), which relaxes DNA transcription supercoils (104, 105). Secondly, doxorubicin generates reactive oxygen species (ROS) damaging cell membranes, DNA and proteins (103, 104) through stimulation of p53-DNA binding; subsequently it initiates caspase signaling and DNA cross-linking (106). Doxorubicin treatment efficacy is often associated with adverse side effects such as nephrotoxicity, hepatotoxicity, sarcopenia, cardiotoxicity (102), and changes in body composition (decreased body weight and lipoatrophy, discussed in section Doxorubicin Toxicity on Adipose Tissue/Adipocytes) (107, 108). These effects contribute toward recurrence as well as metastasis in breast cancer patients, making doxorubicin treatment protocols ineffective and prone to develop treatment resistance (109, 110).

### Obesity and Treatment Resistance

Experimental animal models showed that diet-induced obesity increases tumor development, progression and metastasis with decreased chemotherapeutic efficacy (7, 8, 55, 111–113), specifically in the case of breast cancer (16–18). Additionally, obesity is associated with larger tumor sizes and positive lymph node involvement compared to non-obese breast cancer patients (114, 115).

Human studies also show that obesity is linked to poor clinical outcomes in breast cancer patients treated with chemotherapeutic, hormonal-based chemotherapy agents and radiotherapy (22, 116). Obesity was also associated with lower pathological complete response, disease free survival, clinical benefit rate and worse overall-survival (22). Iwase et al. reported that a high visceral fat area is associated with poor clinical outcomes for patients receiving neo-adjuvant chemotherapy (anthracycline followed by taxane) treatment regimens (19). In fact, treatment protocols for overweight and obese cancer patients includes prescribed lower doses of chemotherapeutic agents to avoid co-morbidities, side effects and adverse toxicities (117). This could also compromise drug efficacy and contribute to the development of treatment resistance and added cytotoxicity (118). However, alterations in dosages cannot clarify all occurrences of treatment resistance in relation to obesity (96, 119, 120).

#### Resistance Mechanisms

Drug resistance can either be classified as *intrinsic (pre-treatment)*, or *acquired* (*post treatment*) (121). Currently, known drug resistance mechanisms include, the evasion of therapy-induced apoptosis, activation of drug transporter proteins and enhanced DNA repair mechanisms, which describe cellular mechanisms (109). Drug resistance can additionally ensue as a result of alterations in pharmacokinetics, drug inactivation and metabolism (109, 121), which can be induced and/or exacerbated in obese states.

##### Cellular mechanisms

Treatment resistance can develop due to the evasion of apoptotic pathways by increased anti-apoptotic protein (*blc-2*) and decreased pro-apoptotic protein (*bax*) expression (122). Adipocytes protect cancer cells from chemotherapeutic agents (i.e., vincristine and daunorubicin) by upregulating anti-apoptotic *bcl-2*, and downregulation of pro-apoptotic *bad* and *pim-2* family members (an oncogene which phosphorylates *bad*) (123). Although the mechanisms by which adipocytes achieved this ‘protection of cancer cells' was not assessed, a recent *in vitro* study identified resistin (mainly secreted from adipose tissue) (124) as a causal factor for acquiring resistance to doxorubicin treatment in both the MCF-7 human breast cancer cell line (estrogen receptor positive) as well as in the MDA-MB-231 human triple negative cell line. Here, doxorubicin induced apoptosis (increased cytochrome-c concentration, cleaved caspase-9, cleaved PARP) in a time and dose dependant manner. Addition of recombinant resistin to the treatment protocol downregulated apoptosis by inducing autophagy (a self-degradation process which cancer cells can utilize to eliminate toxic materials to avoid cell death) (25). Although resistin receptor expression was not assessed, and no supporting evidence for animal or human models were provided, it would be plausible to motivate for more experimental research to investigate potential mechanisms and causal factors involved in acquiring doxorubicin treatment resistance.

Additionally, treatment resistance can also be the result of gene mutations coding for apoptotic proteins (125), for example the mutation in p53 has been associated with acquired resistance to doxorubicin in breast cancer patients, possibly due to inhibition of apoptosis by activating Bax/Bak (pro-apoptotic factors) (125). General-, and central obesity showed a positive association with mutations in p53 of tumor tissue, which was further associated with less favorable tumor characteristics including poorly differentiated and higher nuclear grade tumors (126).

Moreover, modifications in the activation and expression of drug transporter proteins alters drug responses by reducing intracellular drug concentration, which promotes treatment resistance (127). Examples are (i) P-glycoprotein (P-gp), (ii) multi-drug resistance protein-1 (MDR-1), (iii) multi-drug resistance associated protein-1 (MDRP-1), and (iv) breast cancer resistance protein (BCRP), which are ATP-binding cassette (ABC) transmembrane pumps responsible for the elimination of toxic compounds from cells (128, 129). Although normally expressed in healthy tissue, overexpression of P-gp, MDR-1, MDRP-1 and BCRP are present in breast cancer cells in relation to doxorubicin resistance (130–132). P-glycoprotein expression can also be upregulated by inflammation (NFκB), resulting in an altered expression of MDRP-1, which increases the expression of P-gp and consequently modify drug responses. (110, 133).

Adipose tissue is also a source of mesenchymal stem cells which share similar characteristics to tumor-initiating stem cells (134), which can be recruited to the tumor microenvironment to support breast tumor growth and proliferation (135). Tumor-initiating stem cells has the ability to self-renew and/or differentiate, tolerate high levels of DNA damage, increase ABC transmembrane transporter protein expression and induce the synthesis of various cytokines and growth factors (increased IL-6 and C-C motif ligand 5 (CXCL5) levels) (63, 135–137), and therefore may be an alternative treatment resistance mechanism. Elevated leptin concentrations and leptin receptor expression (increased in adiposity) is associated with the promotion of cancer stem cells survival and self-renewal, by inducing JAK2/STAT3 signaling pathways, that increase stem cell renewal transcription factors (NANOG, OCT-4, and SOX-2) expression in breast cancer cells (77, 138).

Leptin, well-known for its role in inflammation and tumorigenesis (74, 139), increases cellular proliferation and angiogenesis (75), and is also associated with higher pathological grade breast cancer tumors (76) and breast cancer treatment resistance (136, 140). However, in contrast leptin also shows anti-cancer effects, by enhancing the anti-proliferative effects of 3′-5′-cyclic adenosine monophosphate (cAMP) elevating agents in breast cancer cells (141). 3′-5′-cyclic adenosine monophosphate is an intracellular second messenger, generated from ATP by adenylate cyclase's (142, 143) and plays a regulatory role in cellular proliferation, apoptosis as well as differentiation, proposed to be induced *via* protein kinase A (144, 145). The utilization of cAMP elevating agents has been explored in pre-clinical models and shows anti-cancer effects i.e., inducing apoptosis (downregulation of Bcl-2 which leads to caspase-3 mediated apoptosis) (146) and cell cycle arrest (146, 147) in breast cancer cells. Additionally, cAMP elevating agents inhibit both cellular proliferation (148), and angiogenesis (decreased VEGF) (149) as well as sensitize breast cancer cells to chemotherapeutic drug treatments (150). Naviglio et al. showed that co-treatment of triple negative breast cancer cells (MDA-MB-231 cells) with leptin and a cAMP elevating agent (forskolin) decreased breast cancer cell proliferation by inhibiting the activation of the ERK signaling pathway (141), which is well-known to be over active in breast cancer cells (151, 152). Interestingly, the authors also showed that leptin enhanced the anti-proliferative effects of cAMP elevating agents, by inducing both apoptosis and cell cycle arrest (141). Additionally, Spina et al. showed that an increase in cAMP levels inhibits leptin-induced migration of breast cancer cells (MDA-MB-231) (153). Recently, Illiano et al. demonstrated that forskolin treatment (cAMP elevating agent) inhibited ERK1/2 activity *via* protein kinase A-mediated inhibition, which induced apoptosis and increased the sensitivity of breast cancer cells (MDA-MB-231 and MDA-MB-468) to doxorubicin treatment (154). This finding is significant since, doxorubicin treatment resistance in breast cancer cells involves the activation of the RAS/RAF/ERK signaling pathway (152, 155). The anti-proliferative interaction between leptin and cAMP elevating drugs might provide potentially new strategies for therapeutic intervention in overweight/obese breast cancer patients (since leptin levels are elevated in overweight/obese patients), who are at risk/prone to develop treatment resistance. However, treatment of breast cancer cells with cAMP elevating agents under obesogenic conditions, is yet to be explored, especially considering cAMP can stimulate lipolysis in adipose tissue (156).

Additional growth factors secreted by adipocytes also implicated in treatment resistance include IGF-1 and IGF-1R (increased systemic bioavailability in adiposity and adipocytes also secrete IGF-1). These growth factors are linked to decreased apoptosis, increased cancer cell proliferation and pro-inflammatory mediator secretion which are directly associated with breast cancer risk and progression (157–160). Upregulation of IGF-1R was associated with poor disease prognosis and chemotherapy resistance through increased expression of MDR-1 and MDRP-1 affecting drug transportation and delivery in cancer cells (161).

Acquired resistance to doxorubicin and docetaxel in breast cancer cells was also attributed to the transfer of microRNA present in exosomes (nanovesicles which mediates cell-cell transfer of DNA, mRNA, microRNA, proteins and lipids) (162). Adipocyte derived exosomes has been associated with increased migration in breast cancer cells (163), immune cell recruitment of macrophages and chronic inflammation (164, 165). Resistance to paclitaxel in ovarian cancer cells was attributed to the transfer of microRNA (miR21) present in adipocyte derived exosomes (166, 167), which downregulated the expression of apoptotic protease activating factor-1, a key protein involved in apoptosome formation (166). In addition, adipocyte derived exosomes increased the invasion of melanoma cancer cells and induced metabolic reprogramming by transferring proteins (ECHA (subunit of *mitochondrial trifunctional protein)* and hydroxyacyl-coenzyme A dehydrogenase), involved in fatty acid oxidation to these cancer cells. In addition, these effects were found to be worsened by obese adipocytes (168). However, evidence on the role of adipocyte and/or obese adipocytes derived exosomes in treatment resistance on breast cancer cells are lacking and therefore motivates experimental models to investigate potential mechanisms and causal factors involved in acquiring doxorubicin treatment resistance.

##### Drug metabolism mechanisms

Adiposity alters chemotherapeutic pharmacokinetics by; (i) increasing drug distribution, (ii) altering drug clearance, and (iii) modifying the drug-protein binding process (169). For example, obesity increases the distribution volume of lipolytic drugs by increasing its' accumulation in excess adipose tissue (169), thereby decreasing exposure of cancer cells to treatment agents. Behan et al. showed that excess adipose tissue could act as a “*shelter*” for protection against treatment toxicity, as cancer cells migrate into adipose tissue (123).

Obesity affects drug clearance *via* the liver, which primarily metabolizes, detoxifies and clears drugs from circulation (170). Hepatosteatosis decreases hepatic microcirculation, whereas the glomerular filtration and tubular secretion, and reabsorption in the kidneys, leads to increased drug clearance (117). Ghose et al. reported that mice fed a high fat diet, showed a decreased expression of key hepatic drug metabolizing enzymes (i.e., CYP3A11, CYP2B10 and CYP2A4), which could be the result of the high levels of pro-inflammatory mediators (IL-1β, IL-6 and TNF-α), increased phosphorylation of JNK and increased activation of NFκB (171). CYP34 activity, has also been found to be increased in a leptin knockout obesity model (172). In addition, the elimination, or the half-life of a drug may also be altered in obese individuals (173). Lastly, obesity is also associated with an increase in alpha-1 acid glycoprotein concentration, which could increase the binding of drugs in the plasma, thereby decreasing its bioavailability (174).

Furthermore, cytarabine, a treatment agent used in acute myeloid leukemia, is only toxic to cancer cells in its phosphorylated form (cytarabine triphosphate) (129). Cancer cells disrupt the phosphorylation reactions by altering the expression of enzymes involved in the metabolic activation of cytarabine i.e., aldo-keto reductase (AKR) and carbonyl reductase (CBR) (122). Sheng et al. showed that adipocytes metabolized daunorubicin (by increasing the expression of daunorubicin-metabolizing enzymes i.e., AKR-1C1, AKR-1C2, AKR-1C3 and CBR-1), which lead to the inactivation of daunorubicin and acquired resistance (175). This could implicate adipocytes/adipose tissue as a co-factor to decrease certain drug concentration in lipid-enriched tumor microenvironments (175). Evidence now also suggest that cancer cells “manipulate” adipocytes in the tumor microenvironment, in order to survive, but also alter drug pharmacokinetics and induce drug resistance by disrupting lipid storage and metabolism (13, 176).

Several mechanisms exist which can result in the modification of drug metabolism, drug transport and the failure of tumor cells to respond to chemotherapeutic drugs, due to overexpression of drug export proteins in cancer cells (169). It should be emphasized that limited evidence, investigating the role of overweight/obesity on pharmacokinetics of the majority of anti-cancer drugs in clinical trials, exists (170, 177). This is mainly attributed to participant inclusion criteria into phase I clinical trials and pharmacokinetic analyses, that exclude patients with co-morbidities, which is highly prevalent in overweight and obese cases (177).

#### Adipocytes in the Tumor Microenvironment: Lipid-Related Mechanisms

Breast cancer cells co-exist in a sophisticated microenvironment with various adjacent cell types including adipocytes, macrophages, fibroblast and endothelial cells (178). Evidence exist on the beneficial roles of fibroblasts, endothelial cells and macrophages in the tumor microenvironment (179–181). The exact role of adipocytes in the breast tumor microenvironment in treatment resistance remains unclear.

The presence of adipocytes in the tumor microenvironment revealed that breast tumor cells utilize adipocytes to their advantage to promote its survival, growth as well as proliferation and metastasis (13, 176). In addition, the presence of adipocytes in the tumor microenvironment also reduces the toxic effects of breast cancer treatment agents (18). For example, Trastuzumab^®;^ treatment (a monoclonal antibody targeting human epidermal growth factor-2) inhibited breast cancer cell growth in the absence of a lipoma. However, this inhibition was hindered in the presence of a lipoma suggesting that adipose tissue/adipocytes may have an impact on resistance to cancer therapy (176).

**Figure 2:**
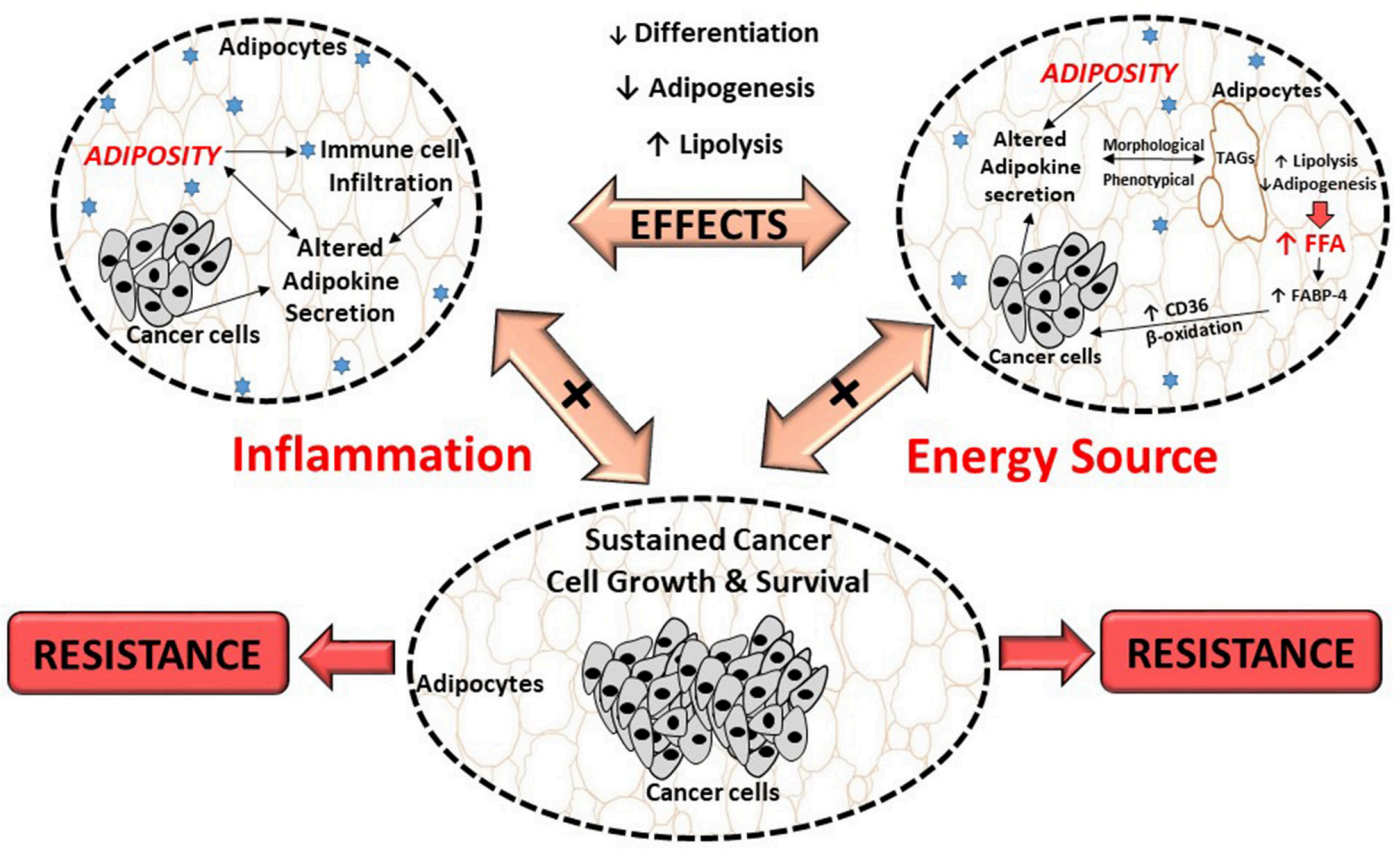
Proposed effects of breast cancer cells on adipocytes and its role in treatment resistance. Breast cancer cells dysregulate metabolic pathways, by altering the secretion of adipokines from adipocytes which results in inflammation. This, could result in morphological and phenotypical changes (delipidation) and thereby increase the release of FFA. These FFA provide energy substrates for cancer cells to sustain its high proliferation demand, contributing to cancer treatment resistance. FABP-4, fatty acid binding protein-4; FFA, free fatty acids; TAGs, triglycerides; CD36, fatty acid translocase.

Adipocytes in the breast tumor microenvironment is characterized by both morphological and phenotypical changes. Histological analysis of human mammary tumor biopsies shows no, or very few adipocytes present (174), with characteristic smaller cell size (14). Adipocytes in the breast tumor microenvironment also display a more fibroblast like morphology known as cancer-associated adipocytes (127, 182). These phenotypical and morphological alterations induce functional changes in adipocytes to yield free fatty acids (FFA) from triglycerides (TG) stored in lipid droplets (Figure 2) (73). This is proposed to be as a result of tumor growth inducing lipolysis in adipocytes, which can result in adipose tissue mass reduction (183).

Previously it was shown that breast cancer cells induce lipolysis by increasing the expression of hormone sensitive lipase (HSL) and adipose triglyceride lipase enzymes in adipocytes (13). It is proposed that adipocyte-derived fatty acids are either used as metabolic substrates for energy (β-oxidation) (184), or stored in lipid droplets and/or membranes within tumors (185), to sustain survival. Fatty acids and its derivatives serve as building blocks for various membrane lipids (i.e., phospholipids and sterol esters) and signaling molecules, both implicated in carcinogenesis and treatment resistance (186–188).

Additional supporting evidence include breast cancer cells increasing exogenous fatty acid uptake and utilization (FFA derived from adipocytes), by altering the expression of various enzymes in fatty acid uptake (i.e., increased fatty acid binding protein-4 (FABP-4) and fatty acid translocase (CD36) expression) (189–191) and β-oxidation (i.e., increased carnitine palmitoyltransferase I expression) (13, 192, 193). In addition, “obese” adipocytes provided higher concentrations of FFA to breast cancer cells to sustain survival and migration (13), however treatment resistance was not assessed in this obese breast cancer model.

Furthermore, adipocytes also provide FFA to breast cancer cells by dedifferentiation and/or inhibition of adipogenesis (13) (Figure 2), evident by adipocytes showing decreased expression of adipogenic markers including peroxisome proliferator-activated receptor-γ (PPAR-γ), FABP-4 and cytosine-cytosine-adenosine-adenosine-thymidine (CCAAT) enhancer binding protein-α (CEBP-α) (14). Breast cancer cells can also alter fatty acid metabolism by increasing *de novo* synthesis of fatty acids, by altering the expression of fatty acid synthase (FAS), acetyl-CoA carboxylase (ACC), and stearoyl CoA-desaturase-1 (SCD-1) enzymes (194–198). The result of this alteration is lipid saturation of cancer cell membranes, which protects against the cytotoxic effects of chemotherapeutic anti-cancer drugs (199). Increased FFA are also stored in tumors in the form of lipid droplets in order to avert lipotoxicity and/or to serve as an energy reserve (200). This is also supported by lipid depositions found in tumors (185), including breast tumors which is considered a characteristic of cancer aggressiveness (201).

It is proposed that the dysregulation of cytokines (increased IL-6, TNF- α and IL−1β), adipokines (increased leptin and decreased Apn and resistin), chemokines, as well as extracellular matrix proteins (collagen IV) from adipocytes (12, 64, 202, 203) (Figure 2), affect the expression of transcription factors involved in lipid metabolism. Examples include, HSL, FABP-4 and CEBP-α (14, 72). The outcome is altered adipocyte-signaling pathways and gene expression in tumor cells which induce stromal cells to produce adipokines (204).

Evidence points toward adipocytes being stimulated by breast tumor cells to increase expression of matrix metalloproteinase-11, a negative regulator of adipogenesis by decreasing pre-adipocyte differentiation and reversing mature adipocyte differentiation (11). Macrophage chemoattractant protein-1 and CCLC-5 may also be active in the host microenvironment promoting survival, invasion metastasis, and unfavorable drug responses. Here, it is proposed that the recruitment of immune cells to the tumor microenvironment, promotes inflammation, stimulating the secretion of matrix metalloproteinase-9 (role in matrix degradation) and evading the host's immune responses (202). Additionally, MCP-1 benefits vascular endothelial cell survival and activate the JAK2/STAT5 and p38 MAPK pathways, inducing angiogenesis (205).

Several groups support the role of cytokines in drug resistance. Estrogen receptor positive sensitive breast cancer cell line, MCF-7, did not express IL-6, however in a drug resistant breast cancer cell line (MCF-7/R) IL-6 was expressed (206, 207). Elevated IL-6 is linked to doxorubicin resistance in breast cancer cells, by increasing cytosine-cytosine-adenosine-adenosine-thymidine (CCAAT) enhancer binding protein (CEBP) activity, which leads to an increased expression of MDRP-1 (206, 208). Additionally, *ex vivo* mature adipocytes significantly increased the proliferation of both mammary cancer cells (MCF-7) and normal mammary cells (184B5) (209). Adipocytes derived from obese patient's diminished Tamoxifen treatment efficacy compared to adipocytes derived from normal weight patients. The authors identified IL-6, TNF-α and leptin as potential mediators (209). In agreement, Incio et al. demonstrated that obesity decreased the efficacy of anti-VEGF treatment in both breast cancer patients and in diet-induced obese mice. The authors proposed that inflammation (increased IL-6) and angiogenesis (increased fibroblast-growth factor-2) in adipocyte dense hypoxic microenvironments within tumors, which can sustain tumor survival (18). However, additional studies are needed to target IL-6 i.e., anti-IL-6R (tocilizumab), in order to identify the exact role of these biomarkers.

To summarize, morphological and phenotypic changes (delipidation), as a result of increased pro-inflammatory cytokines and a deregulated adipokine profile in adipose tissue/adipocytes, may be responsible for breast tumor enhancing effects of adipocytes, providing a potential mechanism for cancer treatment resistance. The role and contribution of adipose tissue/adipocytes in the tumor microenvironment and pathogenesis of breast cancer remains unclear. The exact molecular mechanisms in which breast cancer cells in an obesogenic environment use adipocyte to their physiological advantage to induce treatment resistance, needs to be explored extensively.

## Doxorubicin Toxicity on Adipose Tissue/Adipocytes

Doxorubicin treatment has been shown to negatively impact adipose tissue/adipocytes (26, 29), ranging from metabolic dysfunction to phenotypical changes (27, 106, 210–212), which contribute toward the disruption of adipose tissue homeostasis and lipid storage (Table 1).

**Table 1:** Effect of doxorubicin on adipose tissue and/or adipocytes.

**Model**	**Findings**	**Proposed Mechanism**	**References**
***In vivo*** Rat retroperitoneal adipose tissue Doxorubicin: 15 mg/kg/body weight, 72 hours before sacrifice. ***In vitro*** 3T3-L1 cells (differentiated into mature adipocytes)	*In vivo*: Doxorubicin (10 and100 nM) was toxic to adipocytes, thereby inducing over 90% cellular apoptosis. *In vitro*: Doxorubicin disrupted adipocyte homeostasis: ↓ lipogenesis, ↑ glucose uptake and ↑ lipolysis thereby increasing free fatty acids (FFA) availability.	**Disrupt lipid-related pathways:** The molecular mechanism by which doxorubicin exerts its toxic effects on adipose tissue was still unknown at this point and warranted further investigation	(210)
***In vivo*** Male dawley Sprague rats epididymal fat Doxorubicin: 2.5 mg/kg/body weight, once a week for 11 weeks.	Doxorubicin was found to be a negative regulator of body weight as it resulted in a significant decrease in the body weight of animals on doxorubicin vs. untreated controls. The decrease in body weight was specifically due to a loss in adipose tissue.	**Necrosis:** Adipose tissue undergoes necrosis as a result of chemotherapy. However, there is very limited proposed molecular mechanisms by which doxorubicin exerts its effects on a molecular level and to what extent the damage is and is unclear if it is only due to necrosis or not.	(29)
***In vivo*** Male wistar rats retroperitoneal adipose tissue Doxorubicin: 15 mg/kg/body weight, 72 hours before sacrifice. ***In vitro*** Primary adipocytes isolated from retroperitoneal fat and 3T3-L1 cells (differentiated into mature adipocytes)	Both *in vivo* and *in vitro* models: doxorubicin treatment ↓ adipocyte size compared to controls. *In vivo*: doxorubicin treatment disrupted lipogenesis, i.e., ↓ fatty acid synthase (FAS) and Acetyl-CoA carboxylase (ACC) expression. In addition, primary adipocytes treated with doxorubicin showed a decrease in insulin-stimulated glucose uptake.	**Phenotypical and metabolic dysfunction:** This may have been the result of decreased expression of proteins regulating lipogenesis and therefore decreased lipid storage.	(27)
***In vitro*** Mice Doxorubicin: 8 mg/kg body weight, for 4 weeks.	Doxorubicin treatment resulted in a significant ↓ in bodyweight and serum tricylglyceride (TG) concentration compared to saline treated mice.	**Changes in body composition:** Proposed by authors to be the underlying reason for cardio-dysfunction in this animal model.	(28)
***In vivo*** Male wistar albino rats Doxorubicin: 2 mg/kg/body weight for 7 weeks.	A significant increase in fatty acid binding protein (FABP) concentration was observed in rats treated with doxorubicin compared to control animals,	**Disrupt lipid-related pathways:** Doxorubicin treatment affects markers regulating adipogenesis.	(211)
***In vivo*** 3T3-L1 cells (differentiated into mature adipocytes)	Doxorubicin treatment resulted in the inhibition of adipogenesis i.e., ↑ expression of PPAR-α, and ↓ PPAR-γ and FABP-4 expression in a dose-dependent manner. Adipocytes which over expressed PPAR-γ and were treated with doxorubicin counter acted all the above effects of doxorubicin.	**Disrupt lipid-related pathways:** Doxorubicin acts as an inhibitor of adipogenesis, by being an antagonist to PPAR-γ expression, which may ultimately lead to a lack of fat accumulation.	(26, 105)
***In vitro*** Male wistar rats treated with doxorubicin (15 mg/kg/body weight, 72 h before sacrifice).	Doxorubicin treatment caused a significant ↓ epididymal adipose tissue weight and adiponectin an increase in serum insulin, glucose, FFA concentration levels compared to saline controls. Doxorubicin treatment caused a decreased HOMA-IR (measurement of insulin resistance) and glucose uptake vs. control animals, which is indicative of impaired insulin sensitivity, and these animals displayed insulin resistance, hyperglycaemia, and hyperinsulinemia.	**Metabolic Dysfunction:** These findings were the result of decreased expression of both AMKP and GLUT-4 in skeletal muscle, which was confirmed by the *in vitro* experiments. The authors concluded that doxorubicin treatment caused hyperglycaemia and insulin resistance, mediated by inhibition of AMPK.	(106)
***In vivo*** T2DM mice model (db/db, leptin knockout) treated with doxorubicin (15 mg/kg/body weight, 5 days before sacrifice)	Doxorubicin treatment induced an inflammatory milieu in diabetic muscle by exacerbating a pro-inflammatory microenvironment (upregulating transcription factor HIF-1α, NFκB, and TNF-α) as well as decreasing anti-inflammatory actions (downregulating regulatory molecule AMPK and IL-15). Doxorubicin treatment induced a dysregulation in glycolytic metabolism in diabetic skeletal muscle by upregulating pyruvate dehydrogenase kinase-4 and lactate dehydrogenase and downregulating phosphorylation of ACC.	**Metabolic Dysfunction:** Results suggest that doxorubicin treatment in the context of diabetes may cause an environment, which can worsen diabetes related effects.	(212)

*ACC, Acetyl-CoA carboxylase; AMPK, AMP-activated protein kinase; FABP-4, fatty acid binding protein-4; FAS, fatty acid synthase; FFA, free fatty acids; GLUT-4, glucose transporter-4; HOMA-IR, homeostatic model assessment of insulin resistance; HIF-1α, hypoxia inducible factor-1α; IL-15, interleukin-15; NFκB, nuclear factor kappa B; PPAR-α, peroxisome proliferator-activated receptor-α; PPAR-γ, peroxisome proliferator-activated receptor-γ; TAGs, triglycerides; TNF-α, tumor necrosis factor-α. ↑, Increased; ↓, Decreased*.

**Figure 3:**
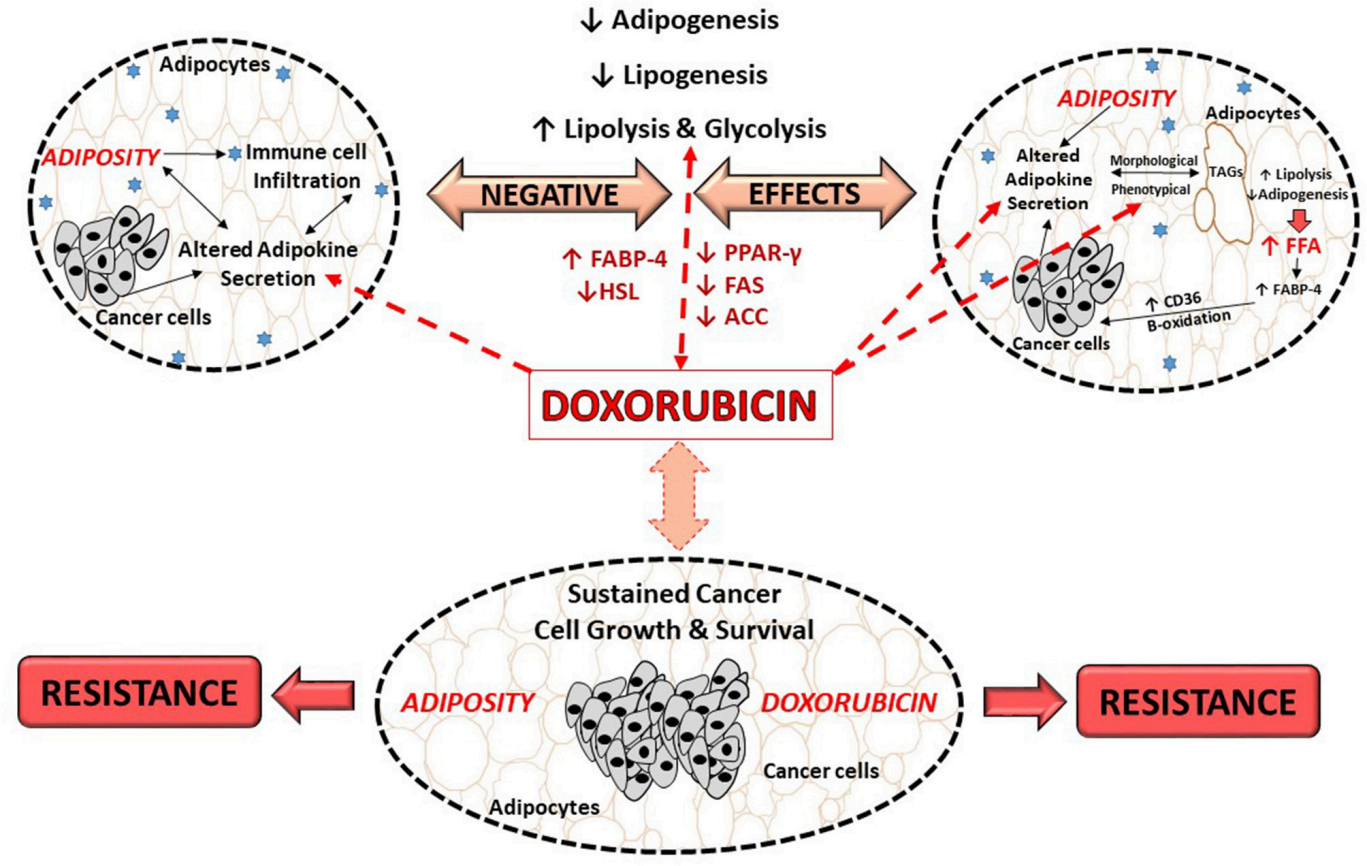
Proposed role of doxorubicin in an obesogenic breast cancer model. ACC, Acetyl-CoA carboxylase; CD36, fatty acid translocase; FFA, free fatty acids; FABP, fatty acid binding protein; FABP-4, fatty acid binding protein-4; FAS, fatty acid synthase; HSL, hormone sensitive lipase; PPAR-γ, peroxisome proliferator-activated receptor-γ; TAGs, triglycerides.

The molecular mechanisms underlying doxorubicin's negative effects on adipose tissue/adipocytes is proposed to involve adipokine dysregulation, which in turn affects factors regulating lipid metabolism pathways. For example, decreasing and/or inhibition of adipogenesis (decreased PPAR-γ and FABP expression) and lipogenesis (decreased FAS expression) as well as the induction of lipolysis (increased HSL expression) (27, 29) (Figure 3). This in turn induces an increase in FFA release as the result of the phenotypical changes (27, 29), thereby disrupting lipid storage. Doxorubicin induced metabolic dysfunction (increased FFA levels), could potentially increase the availability of energy substrates (FFA) for cancer cells to utilize to sustain both its' survival and proliferation demands (26, 27, 29, 106), and thereby indirectly contribute to breast cancer treatment resistance itself. However, it should be stressed that evidence on the effects of doxorubicin on adipose tissue/adipocytes (Table 1) is based on normal functioning adipose tissue/adipocytes, and not on an obesity model, where adipose tissue is dysfunctional.

Evidence on the effects of doxorubicin on adipose tissue/adipocytes in the context of obesity, where adipose tissue is dysfunctional is lacking. In light of this, we proposed that doxorubicin treatment on dysfunctional adipose tissue and/or adipocytes, may further exacerbate the negative effects of obesity itself, toward cancer treatment by further dysregulating adipokines secretion, which in turn affects the factors regulates lipogenesis, adipogenesis and lipolysis, thereby further implicating obesity in the context of breast cancer treatment (Figure 3).

## Future Research and Conclusion

Adipose tissue plays an important physiological role as a metabolically active storage compartment and endocrine organ. A disruption in adipose tissue homeostasis results in potentially serious health and clinical-related outcomes. Obesity induced adipose-dysfunction is associated with an increased risk for breast tumor development and progression.

Obesity is associated with chronic low grade inflammation as a result of adipokine secretion (immune cell infiltration), which results in a sustained inflammatory *milieu*. These inflammatory mediators activate downstream signaling pathways (MAPK and PI3K) in breast cancer cells that favors cancer cell survival (increased proliferation and decreased apoptosis), and contribute to breast cancer development and progression (Figure 3).

Recent evidence also implicate obesity as a causal factor for reduced chemotherapy efficacy, resulting in treatment resistance. Obesity-driven changes may contribute to chemotherapy resistance by altering drug pharmacokinetics, impairing drug metabolism and delivery, and inducing chronic inflammation as well as altering tumor-associated adipocyte adipokine secretion. However, the exact underlying mechanisms by which obesity achieves this remains unclear.

It is suggested that adipose tissue/adipocytes, serve as a potential energy source for cancer cells to sustain their survival thereby promoting cell growth and proliferation (Figure 3). This is especially significant in the case of breast cancer; as adipose tissue is the most abundant tissue type in the breast. Breast cancer cells dysregulate lipid related metabolic pathways i.e., lipolysis, adipogenesis, *de novo* fatty acid synthesis and exogenous lipid uptake by altering the secretion of adipokines by adipocytes, which in turn results in the release of FFA (Figure 3). These fatty acids can then serve as energy substrates for breast cancer cells to sustain its high proliferation rates or can be stored in tumors in the form of lipid droplets and/or in membrane lipids in order to avoid lipotoxicity, which protects against the cytotoxic effects of anti-cancer drugs.

Additionally, doxorubicin treatment itself has also been shown to modify adipose tissue/adipocytes through inhibition of adipogenesis, downregulating lipogenesis, inducing lipolysis, and subsequently disrupting lipid storage. Resulting in phenotypical changes in adipocytes (Figure 3), which in turn produces more “bioavailable” energy substrates (increased FFA), which cancer cells can potentially utilize to sustain survival and proliferation demands and thereby could indirectly contribute to chemotherapeutic treatment resistance (Figure 3).

It should be stressed that studies investigating the effects of doxorubicin on adipose tissue/adipocytes and lipid metabolism in the context of obesity, where adipose tissue is dysfunctional are lacking. We thus propose that doxorubicin treatment in patients with dysfunctional adipose tissue and/or adipocytes, may further exacerbate the tumor promoting effects of obesity itself. This may be achieved by further dysregulating adipokine secretion, which in turn affects lipogenesis, adipogenesis and lipolysis, linking adiposity to breast cancer treatment resistance (Figure 3). It is thus of importance to investigate the effect of doxorubicin in the context of obesity, and how obesity may aggregate factors playing a role in the development of doxorubicin treatment resistance, as there is an increase in the prevalence of breast cancer patients who are either overweight or obese, treated with doxorubicin. Specifically, since, obese and normal weight patients receive the same treatment regimens. Therefore, extensive investigation is needed to elucidate the underlying mechanism by which obesity contributes to treatment resistance.

The role of lipid metabolism in breast cancer also remains understudied as well as the cytotoxic effects of chemotherapeutic drugs on adipose tissue/adipocytes, both of which may contribute to the promotion of breast cancer cell survival and treatment resistance. Therefore, the identification of molecular mechanisms underlying both the effects of a neoplastic state and doxorubicin treatment on adipose tissue, will promote the identification of novel pharmacologic targets as well as the development of appropriate management protocols for adipose tissue driven chemotherapeutic drug resistance as well as doxorubicin related toxicities in order to improve over-all survival of breast cancer patients.

## Author Contributions

IM wrote the first draft of the manuscript. TN, A-ME, and PvJ contributed to critical revision and intellectual input of the manuscript. All authors read and approved the final manuscript.

### Conflict of Interest Statement

The authors declare that the research was conducted in the absence of any commercial or financial relationships that could be construed as a potential conflict of interest.
